# Uncovering the complex genetics of human personality: response from authors on the PGMRA Model

**DOI:** 10.1038/s41380-019-0399-z

**Published:** 2019-03-18

**Authors:** Igor Zwir, Pashupati Mishra, Coral Del-Val, C. Charles Gu, Gabriel A. de Erausquin, Terho Lehtimäki, C. Robert Cloninger

**Affiliations:** 1 Washington University School of Medicine, Department of Psychiatry, St. Louis, MO USA; 2 University of Granada, Department of Computer Science, Granada, Spain; 3 Department of Clinical Chemistry, Fimlab Laboratories, and Finnish Cardiovascular Research Center - Tampere, Faculty of Medicine and Health Technology, Tampere University, Tampere, Finland; 4 Washington University, School of Medicine, Division of Biostatistics, St. Louis, MO USA; 5 University of Texas Rio-Grande Valley, School of Medicine, Department of Psychiatry and Neurology, and Institute of Neurosciences, Harlingen, TX USA; 6 Washington University, School of Arts and Sciences, Department of Psychological and Brain Sciences, and School of Medicine, Department of Genetics, St. Louis, MO USA

**Keywords:** Genetics, Psychology

Following publication of our two articles [1, 2], a critique of the methodology of Phenotype-Genotype Many-to-Many Relations Analysis (PGMRA) [1, 3, 4] questioned the validity of our results from the perspective of polygenic risk scores (PRS) [5]. We appreciate the importance of these questions, and here provide a concise discussion of the assumptions and mathematical constraints of both approaches. We thank this commentator and others who have discussed our articles with us for their thoughtful questions and critiques.

Complex phenotypes present several challenges for genome-wide association studies including the presence of epistasis, pleiotropy, and heterogeneity. We approached these problems in a data-driven fashion to test the hypothesis that the heritability expected from twin studies but unexplained by genetic studies is distributed in heterogeneous partitions of a complex trait, each with distinct genotypic-phenotypic associations. We designed a machine learning algorithm termed PGMRA [1, 3, 4] to identify naturally occurring partitions in the data in an unsupervised fashion. PGMRA first dissects genome-wide data and uncovers a genotypic architecture composed of sets of SNPs shared by subsets of individuals (i.e., SNP sets [3, 6]). Next, phenotypic data are independently organized into natural sets of features such as clinical manifestations [4], voxels of neuroimages [7], or personality traits [1, 2] in a phenomic-like approach [8]. Cross-matching of the two types of sets reveals multiple associations restricted to subgroups of individuals, thereby uncovering the genotypic-phenotypic architecture of a trait and accounting for its distributed genetic risk or propensity.

**Fig. 1:**
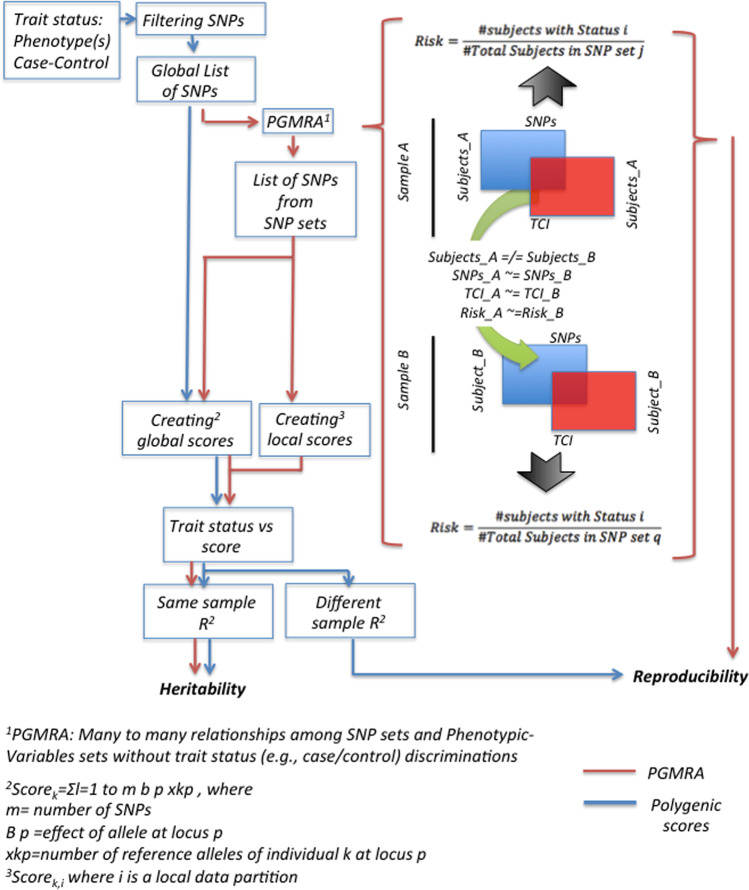
Flow chart describing the common, as well as the different, roads followed by methods developed to build polygenic scores and the PGMRA method

Both approaches, PRS and PGMRA, rely on genome-wide markers (Fig. 1). However, PRS treats these markers as independent variables with additive effects, whereas PGMRA searches for sets of structurally connected markers, which may have interactive effects (epistasis). PRS assumes a global linear association model and relies on increasing sample size to improve performance [9, 10]. In contrast, PGMRA uncovers a family of models (i.e., SNP sets), each of which computes in a local partition of the data. Each model can be represented as either a linear combination of data (as in regression trees) or as a non-linear combination (as in some neural networks) [11]. Therefore, PGMRA uses a more complex model than PRS, focusing on incorporating more phenotypic variables rather than more individuals, but allows the use of smaller samples by reducing multiple comparisons.

PRS algorithms must reduce phenotypes to a single dependent variable because they use a linear supervised model [12]. In contrast, PGMRA uses an unbiased and unsupervised model to consider all possible phenotypic patterns common to a subset of individuals, regardless of their trait status (i.e., does not assign cases and controls a priori). Distinct patterns of phenotypic features can thus be associated with different SNP sets, thereby uncovering heterogeneous subtypes of the trait [1, 2, 4]. Finally, PGMRA incorporates trait status a posteriori to calculate the risk of such associations, and then independently tests the significance of the associations by a SNP-set Kernel Association Test [6, 13].

The validity of the replication procedure used by PGMRA was questioned too [5]. The “gold standard” approach used by PRS evaluates the reproducibility of an association by building a linear classifier trained in a *discovery* sample and testing it in a new sample assuming sample homogeneity [9, 10]. Homogeneity is a strong assumption that should be supported. By contrast, PGMRA uncovers genotypic-phenotypic associations for sample partitions and computes their corresponding risk or propensity post hoc; this process is blindly repeated independently for each new sample without assuming homogeneity within or across samples (Fig. 1). Then, similar genotypic-phenotypic associations across samples with comparable risk/propensity are uncovered using parsimonious models that balance accuracy with model complexity, thereby avoiding overfitting [11, 14, 15].

Inconsistent results obtained from applying PRS to heterogeneous samples [16, 17] has led to the suggestion of averaging scores from multiple samples [18] ignoring, at least in part, the phenotypic heterogeneity of the samples. When there is complexity derived from genetic, cultural, ethnic and environmental heterogeneity, the same global linear model is unlikely to predict across samples, especially when markers have relatively small effect [12, 16, 17]. Models learned independently in diverse samples allow analysis of replication across potentially heterogeneous samples, thereby providing a more stringent test of reproducibility [19, 20].

PRS calculates heritability as an adjusted *R*^2^ from a global linear regression, which additively estimates variance explained by the markers. In the absence of a validated estimator of variance for “sets” of markers [6, 13], PGMRA used a similar approach (Fig. 1). For example, the estimated heritability of character, without controlling for outliers and jackknife resampling, in the Finns sample [1] was 45.67%. A criticism [5] questioned the lack of application of another sampling technique such as cross-validation. As suggested, we applied cross-validation within and across samples (e.g., *R*^2^ of 10 k-fold is 45.05% with SD 0.049) and confirmed the observed results by bootstrapping (1,000 iterations, SE < 1.6%). We also found that the estimates of heritability for character in our paper [1] are conservative: the aggregation of the local variances explained by all SNP sets delivers a higher estimation of heritability (*R*^2^ > 15%) than the 45.67% described above (Fig. 1, unpublished results).

Some suggest that our sample size (2126 + 972 + 902 individuals from 3 cohorts, respectively [1, 2]) has insufficient power, even though others have calculated 80% power at nominal significance to detect heritability with the same sample size [12]. PGMRA computes genotypic-phenotypic associations based on “sets” of genotypes and “sets” of phenotypes, so the number of multiple comparisons are significantly reduced, making PGMRA less greedy of observations than PRS.

The nature of human beings embraces complex functions where every expressed gene may affect the function of any cell and their derived traits of our body in many different ways (many-to-many relationships). Complex traits are expected and known to be influenced by multiple genes acting in concert, not independently [21]. Most of the heritability in gene expression is determined by many genes far apart on the same or different chromosomes [21–23], whose effects are difficult to detect due to their small magnitude (e.g., trans eQTLs effects), as well as co-expressed genes that are vulnerable to decoherence in response to environmental perturbations [24]. PGMRA opens the door to develop new methods to explain complex genotypic-phenotypic relationships, including epistasis, pleiotropy and heterogeneous phenotypes, which present problems for PRS due to its restrictive linear model and doubtful assumption of homogeneity. Use of PGMRA would allow more thorough study of moderate-sized samples by efficient data-driven methods, which can help to bring methods of precision medicine into practice [1–3, 7, 20, 25].
